# Mitoptosis, a Novel Mitochondrial Death Mechanism Leading Predominantly to Activation of Autophagy

**DOI:** 10.5812/hepatmon.6159

**Published:** 2012-08-20

**Authors:** Jaganmohan Reddy Jangamreddy, Marek J. Los

**Affiliations:** 1 Deptartment of Clinical and Experimental Medicine, Integrative Regenerative Medicine Center (IGEN), Division of Cell Biology, Linköping University, Linkoping, Sweden

**Keywords:** Homeostasis, Cytochromes C, DNM1L Protein, Human

Sometimes some members of multicellular organisms need to sacrifice for the good of the whole. Perhaps with the exception of immunomodulatory processes (1, 2), it is the intrinsic death pathway, often triggered by p53 (3, 4, 5), modulated by Bcl2-family members, and executed primarily by caspases that is most commonly employed to trigger cell death (6, 7, 8). Apoptotic or autophagic cell death is triggered by physical insults such as cold (9), natural compounds and their derivatives (10, 11, 12), viruses (13), or even disturbances within the cell cycle (14, 15). Apoptotic cell death is preceded by mitochondrial release of cytochrome c, which leads to increases in cytochrome c in serum (16). Mitochondria have been a cellular guest for millions of years and seamlessly transformed into a major functional cellular organelle. Until the last couple of decades, mitochondria were mainly viewed as powerhouses of the cell but more recent reports have indicated their crucial role in apoptosis, necrosis, and autophagy. Opening of the permeability transition pore in the outer mitochondrial membrane, release of cytochrome c, and formation of apoptosomes is considered the turning point in apoptosis. Further studies showing the cellular localization and phenotypic and mechanistic modulations in mitochondria during cellular homeostasis, stress, and death, support the pivotal role of mitochondrial influenced cellular fate.

**Figure 1:**
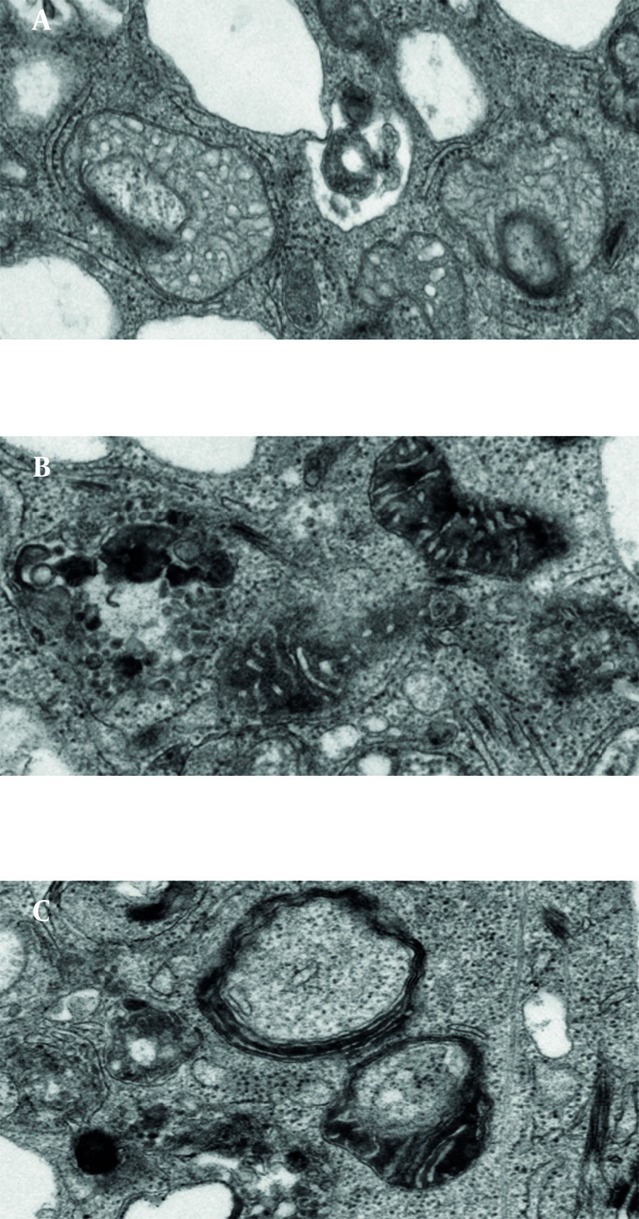
Ultrastructural Forms of Mitoptosis. Mitoptosis was induced in PC3 prostate cancer cells Inner Membrane Mitoptosis (A) and in SKBR3 breast cancer cells by overnight treatment with salinomycin. Inner membrane mitoptosis (A) and outer membrane mitoptosis Outer Membrane Mitoptosis (B) in the apoptotic breast cancer cell and prostate cancer cell lines. We have also observed the third type of mitoptosis, which we have coined mitochondrial matrix mitoptosis Mitochondrial matrix Mitoptosis (C) in which both membranes are degraded with the matrix.

Thus, do mitochondria have the mechanisms to trigger host cell death or is the host directing the mitochondria depending on physiological needs? To what extent are mitochondria autonomous in their function and death? Recent reports about mitochondrial suicide (mitoptosis) and relocation of mitochondria to the nuclear periphery (thread-grain transition) may provide substantial answers to these basic questions. Two very interesting reviews (Skulachev, IUBMB Life 2000, and Skulachev, Apoptosis 2006) by Vladimir P. Skulachev elaborate the fundamental understanding of mitochondrial suicide and the phenomenon of apoptosis and coined the term mitoptosis (17, 18). Mitoptosis takes various forms (Figure 1). Inner membrane mitoptosis may occur, in which only the internal matrix and cristae are degraded while the external mitochondrial envelope remains unaltered, or outer membrane mitoptosis may occur, in which only swollen internal cristae are detected as remnants. Furthermore, the fate of the degraded mitochondria may differ under different experimental conditions. The degraded mitochondria may either become autophagosomes (predominant phenomenon observed in our lab), or mitoptotic bodies, which are extruded from the cell (19).

During “outer mitochondrial membrane mitoptosis”, mitochondria undergo condensation, followed by swelling and fragmentation of cristae. Finally, the outer mitochondrial membrane bursts, and the vesicular remnants of cristae float into the cytoplasm. Mitochondrial swelling can be detected even at the fluorescence microscopy level. At high resolution, mitochondria appear round and swollen, before they disintegrate, rather than typically elongated and bean-shaped. During “inner mitochondrial membrane mitoptosis”, the outer mitochondrial membrane remains intact and the cristae deteriorate. The inner membrane begins to coalesce, followed by rarefaction (loss of density) of the matrix, and finally degradation of cristae. We have often observed a third mixed form of mitoptosis in which mitochondria undergo condensation, followed by swelling and vesicular fragmentation of cristae, similar to “outer mitochondrial membrane mitoptosis”, but instead of disruption of the outer mitochondrial membrane, the mitochondria become engulfed in autophagosomes. Thus, the fate of mitochondria inside stressed cells varies, and the study of mitoptosis in different model systems and the subcellular mechanisms underlying these processes still await conclusions. Mitoptosis occurs primarily due to the loss of membrane potential either because of the apoptotic signal or disruption in the respiratory chain, the inherent inability to synthesize major constituents, and failure to take up the nuclear-coded mitochondrial matrix proteins due to the loss of own membrane potential (18). Thus, it can be inferred that the apoptotic stimulus triggering loss of mitochondrial membrane potential is the major factor initiating mitoptosis. However, the initial apoptotic signal increases mitochondrial membrane potential during the early steps of apoptosis, eventually leading to loss of membrane potential. This initial increase in membrane potential is thought to be due to the ATP dependency of apoptosis, hence, the distantly located mitochondria (resulting from mitochondrial fission or thread-grain transition) need to be transfered to the nuclear surroundings to release apoptotic factors for nuclear transfer; thus, amplifying programmed cell death (18). This observation suggests that mitochondrial dysfunction and the production of reactive oxygen species (ROS) are major factors triggering mitoptosis. Such observations are further supported by studies using mitochondrial respiratory chain uncouplers and mitochondrial poisons in which overproduction of ROS could be observed without reductions in cellular ATP levels leading to mitoptosis (20). The specific removal of dysfunctional or ROS-overproducing mitochondria during apoptosis or mitoptosis is believed to be achieved by autophagy either by autophagosome formation (mitophagy) or by the formation of mitoptotic bodies that are subsequently released into the extracellular environment (19). The elimination of dysfunctional mitochondria is further supported by studies of cells treated with staurosporin, a common drug used to induce apoptosis, and by the use of pan-caspase inhibitors in which cells survive but lose their mitochondria (21). More recent studies on PINK1 and Drp1 in neural diseases suggest that dysfunctional mitochondria trigger autophagy and, thus, are eliminated (22). Thus, suggesting that mitochondrial dysfunction is a good enough reason for eliminating mitochondria and as Dr. Skulachev says, mitochondria follow the samurai’s law; “it’s better to die than to be wrong”.
